# Cross-National Comparisons of Internalizing Problems in a Cohort of 8952 Adolescents from Five European Countries: The EU NET ADB Survey †

**DOI:** 10.3390/children10010008

**Published:** 2022-12-21

**Authors:** Georgia Drosopoulou, Foteini Vlasopoulou, Eleni Panagouli, Androniki Stavridou, Alexia Papageorgiou, Theodora Psaltopoulou, Maria Tsolia, Chara Tzavara, Theodoros N. Sergentanis, Artemis K. Tsitsika

**Affiliations:** 1 MSc “Strategies of Developmental and Adolescent Health”, School of Medicine, National and Kapodistrian University of Athens, 115 27 Athens, Greece; 2 Department of Clinical Therapeutics, School of Medicine, National and Kapodistrian University of Athens, 115 28 Athens, Greece; 3 Department of Public Health Policies, School of Public Health, University of West Attica, 11 521 Athens, Greece

**Keywords:** anxiety, depression, Youth Self Report, internalizing problems, risk factors, cross-cultural

## Abstract

Various factors such as family relationships, socioeconomic indicators, socio-cultural aspects, mental health problems and demographic variables, have been associated with internalizing problems in adolescence. The purpose of this study was to examine the prevalence of internalizing problems in adolescents from five European countries in comparison with risk factors. Using data from the European Network for Adolescent Behavior (EU NET ADB) survey, a cross-sectional school-based study of adolescents (from 14 to 17.9 years) was conducted. Data were retrieved from self-report questionnaires derived from Greece, Spain, the Netherlands, Iceland and Romania. Multiple logistic regression analysis overall and by country was held through estimation of odds ratios (OR) and 95% confidence intervals (95% CI). A total of 8952 adolescents participated in the study. According to the results, Greece (7.6%) and Romania (2.4%) presented with the lowest rates of internalizing problems, while Iceland presented the highest (11.1%). Internalizing problems were associated with lower school grades (adjusted OR = 1.21, 95% CI: 1.08–1.36), while the higher education level of parents was associated with lower odds of internalizing problems (adjusted OR = 0.75, 95% CI: 0.62–0.92). In country-specific analyses, factors that affected the likelihood of internalizing problems were gender, age, maternal and paternal unemployment. Variations detected in adolescents’ internalizing problems were determined by the socio-cultural context of each country. Cultural differences should be addressed thoroughly in further research, in order to better understand and intervene in aspects of internalizing problems in adolescence.

## 1. Introduction

Adolescence represents a pivotal developmental period which is challenging, due to various changes imposed on the transition context of this age. The well-being of adolescents has been investigated under various methodological approaches; some common indicators correlating with well-being are socio-economic factors, family history, history of mental disorders, gender, relationships, school environment, environmental and cultural factors [1,2].

The terms internalization and externalization are widely used to describe two major categories of adolescent and children’s mental health problems that reflect behavioral, emotional, and social difficulties [3]. Internalizing and externalizing problems were first introduced to describe the categories of problems that stemmed from factor analysis [4]. Internalizing problems fall into four main types of specific syndromes, disorders, or symptom groups, namely depression, anxiety, social withdrawal and physical problems [5,6].

Internalizing problems in adolescence have been thoroughly examined by many researchers, in association with gender, age and other demographic variables. Gender has emerged as an important predictor of internalizing problems, with female adolescents being at least twice as likely to develop symptoms of anxiety and depression in adolescence, a pattern that persists in adulthood [7,8]. In a sample of 27,206 individuals, Rescorla et al. (2006) examined the effects of culture, gender, and age on Achenbach youth self-report (YSR) scores from 24 countries, and found that girls scored significantly higher than boys in internalizing problems in most countries [9].

In the same study, adolescents aged 14–16 reported more problems than adolescents aged 11–13 years. Particularly in terms of internalizing problems, older adolescents had higher scores. This is consistent with research in various aspects of adolescent mental and physical health, showing that older adolescents are generally more likely to suffer from difficulties concerning life satisfaction, physical and mental well-being [1,10].

Low socioeconomic income has also been associated with an increased risk for internalizing disorders, and less for externalizing disorders [11,12]. Recently, Philipp et al. (2018) showed that adolescents from low-income or single-parent families are at an increased risk of internalizing problems. As expected, better family affluence has been correlated with better well-being outcomes, due to the fact that children have access to healthier lifestyle habits and are less exposed to feelings of insecurity, anxiety and unfavorable social comparison [1,13].

In a cross-cultural perspective, increase of income inequality within a country has been associated with decline in child well-being [1,14]. Moreover, the cultural norms and values of each country, the individualistic or collectivistic orientation, and the historical background display a major influence in the way well-being and psychosocial problems are perceived by the citizens [1,14]. According to Pace et al. (2016), as far as the European framework is concerned, there are different levels of identification with European identity for each country, depending on its unique history and social context. This diversity expands to aspects of well-being, such as self-perception, sense of identity, autonomy and life satisfaction [15].

The present study aims at investigating the prevalence and risk factors for internalizing disorders in adolescents from five European countries, in the context of the European Network for Addictive Behavior (EU NET ADB) survey.

## 2. Materials and Methods

### 2.1. Procedure and Participants

The EU NET ADB study was originally conducted in seven European countries (Germany, Greece, Iceland, The Netherlands, Poland, Romania and Spain), in a total sample of 13,708 adolescents. It took place between October 2011 and May 2012, using a common protocol for all countries. The methodology received ethical approval in each participating country [16]. The forms completed by the students’ guardians ensured the confidentiality and anonymity of the students’ personal data prior to conducting the research. The written consent of the students’ parents was a prerequisite. The response rate, as expressed in the percentages of enrolled students, was very high in all countries (from 95.0% in Spain to 86.7% in Romania), with the exception of Iceland (62.9%).

The sampling was clustered, with the selection of a pooled sample of adolescents attending 9th and 10th grade in each country. The primary unit from which the sample was drawn is the classroom and official national lists were used as sampling frames, which were sorted by region (following the classification of the European Union’s NUTS system) and population density. Next, a random sample of 100 classes was selected in each country to obtain a sample size of 2000 adolescents. These classes were systematically selected from the list, starting from a random starting point. As a result of these processes, each adolescent was equally likely to be selected for the sample, and all students enrolled in the selected classes were eligible to participate in the research.

In the current study, the total sample consisted of 8952 adolescents. Germany and Poland were excluded from the current analysis, because the Youth Self Report (YSR) data were not available. As a result, the data of this analysis include five countries: Greece, the Netherlands, Romania, Spain and Iceland. In Romania, however, the data regarding the YSR subscales were not available for administrative reasons, but the total internalizing scale was calculated in the analysis.

### 2.2. Measures

#### 2.2.1. Youth Self Report Questionnaire

The YSR is a self-report questionnaire, completed by adolescents aged 11 to 18 years [17]. This tool is part of the Achenbach System for empirically documented evaluation. The Achenbach system is a comprehensive set of tools for assessing the abilities, adaptive functionality, and behavioral problems in children, adolescents and adults [18].

#### 2.2.2. Demographic Questionnaire

This makeshift questionnaire was created for the purposes of the EU NET ADB research program. Participants were invited to complete their gender, date of birth, place of birth and overall academic performance in 2010–2011. They were then asked to complete the marital status of the parents (marriage/cohabitation/separated/single parent) and the educational level of the parents (low/medium vs. high). The educational level, which acts as an indicator of socioeconomic status, was defined as the highest of the levels for both parents. They were also asked to complete for each parent their age and occupational status (e.g., employment status, unemployed, retired, housekeeper, student, etc.). Participants answered if they have siblings and filled in their number.

### 2.3. Statistical Analysis

Mean values (standard deviation = SD) were used to describe the numeric variables. Absolute (N) and relative (%) frequencies were used to describe the categorical variables. Pearson’s Chi-squared tests were used to compare percentages at the univariate analysis. Multiple logistic regression analysis was used to examine independent risk factors related to internalizing problems overall, as well as separately in each country. The presence of internalizing problems was set as the dependent variable; risk factors (independent variables) were: gender, age, parental educational level, parental marital and occupational status, siblings, the previous year’s low school grades (1–14.9 vs. 15–20), paternal age and maternal age. All tests were corrected for their complex sample design. Significance levels were two-sided and statistical significance was set at 0.05. SPSS 22.0 (IBM Corp. Armonk, NY, USA) was used for the statistical analysis.

## 3. Results

As shown in Table 1, of the total sample, 4689 (52.4%) participants were females and 4263 (47.6%) participants were males. Participants ranged in age from 14 to 17.9 years. Parents’ educational attainment was high (higher education graduates) in 63.6% of participants. However, a significant proportion of students (43%) had an unemployed father or mother. In terms of marital status, for the majority of participants (81.2%), parents were in a marriage or living together. 88.4% of students said they had siblings. In 47.8% of students, the school performance during the last academic year corresponded to more than 15/20.

Table 2 shows the proportion of participants in the clinical spectrum (>98th percentile) for internalizing problems by country. With regard to anxiety or depression symptoms, Iceland (6.9%) showed the highest rates of clinical problems, followed by the Netherlands (4.4%) and Spain (4.1%), while Greece had the lowest rate of clinical problems (1.5%). The prevalence of social withdrawal/depression at the clinical level was lowest in Greece (1.8%) and Iceland (2.2%), followed by the Netherlands (3.1%) and Spain (3.3%). In addition, somatic complaints showed the highest rate in Iceland (9.3%) and the lowest in Greece (2.7%). Regarding the total internalizing problems, Iceland showed the highest rates (11.1%), followed by the Netherlands (10.7%), whereas Greece (7.6%) and Romania (2.4%) showed the lowest rates.

Table 3 presents the results of multiple logistic regression analysis in the total cohort. Participants from Spain were more likely to report anxiety/depression than those from Greece (adjusted OR = 3.08, 95%CI: 1.59–5.98). Participants from Iceland were more likely to have clinical scores in internalizing problems (adjusted OR = 1.52, 95%CI: 1.08–2.14), anxiety/depression and somatic complaints subscales, than Greeks. However, participants from Romania were less likely than Greeks to report internalizing problems (adjusted OR = 0.24, 95%CI: 0.15–0.39). Adolescents aged 16–17.9 years were more likely to report somatic complaints than adolescents aged 14 to 15.9. Higher education level of parents was found to be associated with lower rates of internalizing problems overall (adjusted OR = 0.75, 95%CI: 0.62–0.92). The previous year’s low school grades were associated with internalizing problems overall (adjusted OR = 1.21, 95%CI: 1.08–1.36), as well as with withdrawal/depression and somatic complaints subscales.

Regarding country-specific analyses, in Greece (Table 4) adolescents whose parents had a higher education level were less likely to report internalizing problems than those whose parents had a low or middle education level. The previous year’s low school grades were associated with internalizing problems overall in Greece as well as in Iceland (Table 4 and Table 5, respectively) and subscales in Spain (Table 6). Mother’s age was found to be associated with a higher rate of internalizing problems subscales in Greece and anxiety/depressed problems for Spain (Table 6 and Table 7, respectively), whereas an inverse correlation with somatic complaints was noted in Iceland. Mother’s and/or father’s unemployment was associated with withdrawal/depression in Greece, but an inverse association with somatic complaints arose in Spain.

Boys were more likely to suffer from internalizing problems marginally in Spain and significantly in Romania (Table 6 and Table 7, respectively), and specifically withdrawal and somatic complaints for Spain. On the other hand, boys in Iceland were less likely to express internalizing problems than girls (Table 5). Finally, in the Netherlands (Table 8), older adolescents were more likely to report internalizing problems and somatic complaints versus their younger peers.

## 4. Discussion

The importance of this study rests on the large sample of European adolescents from five countries and the associations between the internalizing problems of this population with the country to which they belong, as well as several socio-demographic variables. Research from various scientific fields has stressed the fact that socioeconomic factors are fundamental determinants of the collective health and well-being [1,2,14]. Adolescents and young people are affected by such factors in different ways, that can be understood in a multi-dimensional perspective concerning cultural norms and beliefs, social support, family support, health conditions and, generally, quality of life aspects [1,2].

According to the results of this study, the proportion of adolescents being at a clinical range of internalizing problems was relatively low for Greece. Iceland, on the other hand, had very high rates in anxiety/depression, somatic complaints and internalizing problems overall. This finding is particularly interesting because, in an apparent contradiction, Iceland has been characterized as one of the happiest nations in Europe [19]. At the same time, however, it has one of the highest rates of depressive symptoms and antidepressant consumption among European countries [20]. Different hypotheses could support this finding, including a rise in the awareness and diagnosis of depression, which may affect the way participants self-report such symptoms [20], seasonal affective disorder which has been associated with younger individuals [19], or the economic crisis in 2008 that has unsettled some former balances in the country [20].

Regarding Greece, the low proportions in clinical scores–despite the vast economic crisis of that period–bring into consideration some collectivist cultural characteristics, along with family support that functions as a support network. These characteristics have been found to be protective factors against depression and stress [21], and are associated with better levels of well-being when citizens are confronted with unemployment or poverty [22] compared to more individualistic societies. Additionally, it has been found that life satisfaction in adolescents is lower in countries that experience less equal income distribution. In particular, adolescents who tend to make unfavorable self-judgments through the impact of social comparison [1] are more likely to feel insufficient in a non-equal environment. In addition, Spanish adolescents were more likely to be anxious/depressed than Greeks. This finding reveals some further disparities among European countries that are detected even in countries with similar cultural characteristics, namely Mediterranean in this case.

Older adolescents were more likely to express somatic complaints than younger ones. This finding is relevant with previous research showing that mental disorders, body image dissatisfaction and risk-taking behaviors concerning the body, weight control attempts and unhealthy habits are more common in older adolescents [10]; the association was particularly evident in the Netherlands.

Consistent with previous findings, adolescents whose parents had a higher educational level were less likely to report internalizing problems than those with a low/middle educational level. Indeed, a high educational level has been found to be associated with higher income, family affluence and better levels of well-being [1].

The previous year’s low school grades were associated with internalizing problems, withdrawal/depression symptoms and somatic complaints. In line with our finding, previous research has also underlined the association between low performance and externalizing problems, while high performance was considered to be a protective factor in students’ well-being [23]. Other studies, however, have highlighted the negative aspects of self-pressure for achievement, high levels of discipline, or self-criticism in students who perform well at school [24,25].

In Greece, a high parental educational level was also associated with less clinical internalizing problems. Another interesting finding showed than Greek adolescents whose mother and/or father was unemployed were more likely to report withdrawal/depression, in line with the fact that unemployment of parents has been found to be one of the possible indicators for low levels of well-being [1,2].

However, in Spain, an inverse pattern arose, namely lower rates of somatic complaints in presence of an unemployed parent; this finding can be partly attributed to the fact that long hours of work of both parents certainly affect family life and relationships, and might increase feelings of loneliness and anxiety in adolescents who are more vulnerable or in need of certain support from their parents [26].

Finally, in Greece, adolescents with older mothers were more likely to report more clinical problems in the anxious/depressed, withdrawn/depressed and somatic complaints scale. Older maternal age was associated with the anxious/depressed scale in Spain, as well. On the other hand, an inverse association between maternal age and somatic complaints was noted in Iceland. The topic of maternal age seems multifaceted as it has been associated with some health risk factors for children, but also with positive outcomes as far as social factors are concerned [27].

Concerning the gender variable, in Iceland boys were less likely to report internalizing problems than girls. This is consistent with a lot of previous findings mentioned above connecting internalizing problems with girls rather than with boys. Surprisingly, in Spain boys were found more likely to report problems in the withdrawn/depressed and somatic complaints scale and, in Romania, boys were also more likely to report internalizing problems than girls.

The most important strength of this study is the cross-cultural comparison of risk factors associated with internalizing problems in adolescents. The findings of the study highlighted the variations in patterns of health and its socio-cultural determinants among countries. They also add some useful data to the limited research on cross-cultural aspects of adolescent well-being in Europe. Methodologically, the anonymity of self-reporting and random sampling limited the risk for reporting and selection biases.

Some of the limitations of this study included the availability of data from Romania, and the cross-sectional nature of the study that makes it difficult to establish any cause and effect associations between the variables involved. Although the research of the main risk factors was established, factors such as differentiation in location settings or climate change may play an important role that could be part of future research. Furthermore, the response rate in Icelandic adolescents was adequate, but could be improved. Finally, self-reporting itself could involve bias related to the subjective perception of the participants or social desirability issues that could influence their answers.

## 5. Conclusions

The results of this study underline the diversity that characterizes cultures in the European framework, and provide current research with some guidelines towards a deeper understanding of adolescents’ well-being in the cultural and social context of each country. Such an understanding of the uniqueness of each context, as well as of the similarities among them, is crucial in order to evaluate the well-being of the adolescent population and, consequently, establish effective preventive and therapeutic policies.

## Figures and Tables

**Table 1:** Sample characteristics (N = 8952).

Variables	N (%)
Sample	8952 (100)
Country	
Greece	1967 (18.0)
Spain	1980 (18.1)
Romania	1830 (16.7)
Netherlands	1249 (11.4)
Iceland	1926 (17.6)
Gender	
Female	4689 (52.4)
Male	4263 (47.6)
Age	
14–15.9	5337 (59.6)
16–17.9	3615 (40.4)
Parental educational level	
Low/Middle	2823 (36.4)
High	4922 (63.6)
Parents Married/Living together	7126 (81.2)
Previous Year Grades	
18–20	1340 (16.3)
15–17.9	2588 (31.5)
12–14.9	2648 (32.2)
10–11.0	1402 (17.0)
1–9	245 (3.0)
Mother and/or father unemployed	3787 (43.0)
Siblings	7849 (88.4)
Father’s age. mean (SD)	46.7 (5.8)
Mother’s age mean (SD)	43.5 (5.3)

**Table 2:** Proportion of adolescents being at clinical range (>98th percentile) of internalizing problems by country.

	Greece	Spain	Romania	Netherlands	Iceland	*p*
	N (%)	N (%)	N (%)	N (%)	N (%)	
Anxious/depressed						
Normal/Borderline	1857 (98.5)	1887 (95.9)	N/A	1086 (95.6)	1770 (93.1)	<0.001
Clinical	25 (1.5)	80 (4.1)	N/A	50 (4.4)	131 (6.9)	
Withdrawn/Depressed						
Normal/Borderline	1658 (98.2)	1746 (96.7)	N/A	1101 (96.9)	1862 (97.8)	0.062
Clinical	30 (1.8)	59 (3.3)	N/A	35 (3.1)	42 (2.2)	
Somatic complaints						
Normal/Borderline	1836 (97.3)	1899 (96.5)	N/A	1081 (95.2)	1714 (90.7)	<0.001
clinical	50 (2.7)	68 (3.5)	N/A	55 (4.8)	175 (9.3)	
Internalizing Problems						
Normal/Borderline	1742 (92.4)	1764 (89.7)	1778 (97.6)	1015 (89.3)	1692 (88.9)	<0.001
Clinical	144 (7.6)	203 (10.3)	43 (2.4)	121 (10.7)	212 (11.1)	

**Table 3:** Results from multiple logistic regression analysis with clinical internalizing problems (in all subscales and in total) as the dependent variable in the total sample. Bold cells denote statistically significant associations.

	Internalizing Problems Normal/Borderline vs. Clinical		Anxious/Depressed Normal/Borderline vs. Clinical		Withdrawn/Depressed Normal/Borderline vs. Clinical		Somatic Complaints Normal/Borderline vs. Clinical	
	OR (95% CI) +	*p*	OR (95% CI) +	*p*	OR (95% CI) +	*p*	OR (95% CI) +	*p*
Country								
Greece (reference)								
Spain	1.3 (0.941.79)	0.108	**3.08 (1.59–5.98)**	**0.001**	1.11 (0.54–2.27)	0.773	0.85 (0.51–1.41)	0.530
Netherlands	0.95 (0.59–1.54)	0.846	2.13 (0.9–5.07)	0.086	0.65 (0.25–1.67)	0.368	0.83 (0.44–1.55)	0.559
Iceland	**1.52 (1.08–2.14)**	**0.017**	**5.88 (2.88–11.99)**	**<0.001**	0.65 (0.27–1.56)	0.335	**2.66 (1.59–4.45)**	**<0.001**
Romania	**0.24 (0.15–0.39)**	**<0.001**	N/A		N/A		N/A	
Gender								
Female (reference)								
Male	1.01 (0.83–1.22)	0.933	1.35 (0.98–1.86)	0.069	1.53 (0.97–2.44)	0.069	1.22 (0.9–1.65)	0.197
Age								
14–15.9 (reference)								
16–17.9	1.15 (0.92–1.43)	0.210	1.11 (0.77–1.59)	0.567	0.84 (0.53–1.34)	0.467	**1.61 (1.18–2.18)**	**0.003**
Parental educational level								
Low/Middle (reference)								
High	**0.75 (0.62–0.92)**	**0.007**	1.08 (0.77–1.50)	0.657	0.71 (0.46–1.12)	0.142	0.94 (0.7–1.28)	0.705
Parents married/living together								
No (reference)								
Yes	0.78 (0.61–1.00)	0.055	0.87 (0.59–1.28)	0.472	0.87 (0.48–1.60)	0.664	0.78 (0.57–1.06)	0.116
Previous Year Low Grades	**1.21 (1.08–1.36)**	**0.001**	1.15 (0.94–1.41)	0.187	**1.70 (1.25–2.30)**	**<0.001**	**1.45 (1.23–1.72)**	**<0.001**
Mother and/or father unemployed								
No (reference)								
Yes	1.13 (0.93–1.39)	0.222	1.14 (0.83–1.57)	0.407	1.15 (0.75–1.78)	0.520	0.93 (0.68–1.28)	0.672
Siblings								
No (reference)								
Yes	1.05 (0.76–1.46)	0.761	2.00 (0.95–4.21)	0.068	1.21 (0.57–2.54)	0.620	0.92 (0.56–1.52)	0.739
Father’s age	1.01 (0.98–1.03)	0.656	1.01 (0.96–1.05)	0.784	0.98 (0.92–1.04)	0.469	0.98 (0.94–1.03)	0.492
Mother’s age	1.01 (0.98–1.04)	0.585	1.04 (0.98–1.1)	0.210	1.05 (0.96–1.14)	0.281	1.01 (0.95–1.06)	0.799

+—Odds Ratio (95% Confidence Interval).

**Table 4:** Results from multiple logistic regression analysis with clinical internalizing problems (in all subscales and in total) as the dependent variable for Greece. Bold cells denote statistically significant associations.

	Internalizing Problems Normal/ Borderline vs. Clinical		Anxious/Depressed Normal/ Borderline vs. Clinical		Withdrawn/Depressed Normal/ Borderline vs. Clinical		Somatic Complaints Normal/ Borderline vs. Clinical	
	OR (95% CI) +	*p*	OR (95% CI) +	*p*	OR (95% CI) +	*p*	OR (95% CI) +	*p*
Gender								
Female (reference)								
Male	0.77 (0.50–1.19)	0.240	1.38 (0.48–3.92)	0.547	1.01 (0.45–2.25)	0.986	1.61 (0.7–3.71)	0.264
Age								
14–15.9 (reference)								
16–17.9	1.08 (0.73–1.61)	0.692	0.63 (0.2–2.01)	0.429	1.57 (0.68–3.59)	0.284	1.26 (0.65–2.44)	0.499
Parental educational level								
Low/Middle (reference)								
High	**0.57 (0.39–0.85)**	**0.006**	0.55 (0.22–1.35)	0.187	0.41 (0.15–1.10)	0.076	0.51 (0.25–1.05)	0.067
Parents married/living together								
No (reference)								
Yes	0.88 (0.46–1.66)	0.682	0.55 (0.14–2.16)	0.391	1.73 (0.38–7.96)	0.477	0.94 (0.35–2.53)	0.905
Previous Year Low Grades	**1.33 (1.02–1.74)**	**0.038**	1.77 (0.89–3.52)	0.103	1.60 (0.94–2.73)	0.083	**1.76 (1.21–2.58)**	**0.004**
Mother and/or father unemployed								
No (reference)								
Yes	1.34 (0.90–1.98)	0.148	1.86 (0.63–5.44)	0.256	**2.52 (1.00–6.36)**	**0.050**	1.15 (0.61–2.17)	0.661
Siblings								
No (reference)								
Yes	1.06 (0.54–2.09)	0.862	1.5 (0.20–11.14)	0.688	3.25 (0.41–25.82)	0.262	1.2 (0.37–3.88)	0.758
Father’s age	0.98 (0.94–1.03)	0.440	0.97 (0.89–1.06)	0.549	0.97 (0.9–1.05)	0.450	0.94 (0.88–1.01)	0.112
Mother’s age	1.04 (0.98–1.11)	0.214	**1.13 (1.03–1.25)**	**0.010**	**1.14 (1.05–1.23)**	**0.002**	**1.14 (1.06–1.24)**	**0.001**

+—Odds Ratio (95% Confidence Interval).

**Table 5:** Results from multiple logistic regression analysis with clinical internalizing problems (in all subscales and in total) as the dependent variable *for Iceland*. Bold cells denote statistically significant associations.

	Internalizing Problems Normal/ Borderline vs. Clinical		Anxious/Depressed Normal/ Borderline vs. Clinical		Withdrawn/Depressed Normal/ Borderline vs. Clinical		Somatic Complaints Normal/ Borderline vs. Clinical	
	OR (95% CI) +	*p*	OR (95% CI) +	*p*	OR (95% CI) +	*p*	OR (95% CI) +	*p*
Gender								
Female (reference)								
Male	**0.69 (0.50–0.94)**	**0.019**	1.05 (0.69–1.62)	0.804	1.59 (0.47–5.34)	0.449	0.75 (0.47–1.20)	0.223
Age								
14–15.9 (reference)								
16–17.9	1.09 (0.60–2.00)	0.772	1.42 (0.81–2.51)	0.216	0.98 (0.34–2.82)	0.974	**1.72 (1.01–2.91)**	**0.046**
Parental educational level								
Low/Middle (reference)								
High	1.11 (0.73–1.69)	0.634	1.27 (0.76–2.10)	0.355	0.94 (0.38–2.36)	0.901	1.17 (0.69–1.97)	0.555
Parents married/living together								
No (reference)								
Yes	0.76 (0.53–1.10)	0.148	1.09 (0.67–1.77)	0.731	1.05 (0.34–3.25)	0.928	0.99 (0.66–1.46)	0.941
Previous Year Low Grades	**1.26 (1.00–1.58)**	**0.050**	1.02 (0.76–1.37)	0.898	2.12 (0.96–4.71)	0.064	**1.48 (1.17–1.87)**	**0.002**
Mother and/or father unemployed								
No (reference)								
Yes	1.28 (0.86–1.91)	0.222	1.43 (0.94–2.16)	0.089	1.01 (0.49–2.07)	0.980	1.12 (0.70–1.79)	0.633
Siblings								
No (reference)								
Yes	1.27 (0.36–4.52)	0.709	2.49 (0.30–20.9)	0.394	1.08 (0.13–9.17)	0.946	0.54 (0.20–1.47)	0.221
Father’s age	1.02 (0.97–1.07)	0.403	1.01 (0.96–1.06)	0.774	1.01 (0.96–1.07)	0.645	1.02 (0.97–1.08)	0.451
Mother’s age	0.98 (0.92–1.04)	0.442	1.00 (0.94–1.06)	0.989	1.01 (0.88–1.15)	0.914	**0.94 (0.88–1.00)**	**0.041**

+—Odds Ratio (95% Confidence Interval).

**Table 6:** Results from multiple logistic regression analysis with clinical internalizing problems (in all subscales and in total) as the dependent variable *for Spain*. Bold cells denote statistically significant associations.

	Internalizing Problems Normal/ Borderline vs. Clinical		Anxious/Depressed Normal/ Borderline vs. Clinical		Withdrawn/Depressed Normal/ Borderline vs. Clinical		Somatic Complaints Normal/ Borderline vs. Clinical	
	OR (95% CI) +	*p*	OR (95% CI) +	*p*	OR (95% CI) +	*p*	OR (95% CI) +	*p*
Gender								
Female (reference)								
Male	1.44 (0.97–2.14)	0.068	1.52 (0.77–3)	0.220	**2.80 (1.21–6.48)**	**0.016**	**2.43 (1.10–5.37)**	**0.028**
Age								
14–15.9 (reference)								
16–17.9	1.04 (0.69–1.57)	0.846	0.97 (0.49–1.95)	0.941	**0.42 (0.19–0.92)**	**0.031**	1.24 (0.64–2.41)	0.525
Parental educational level								
Low/Middle (reference)								
High	0.72 (0.48–1.06)	0.090	1.10 (0.61–1.98)	0.747	1.05 (0.47–2.38)	0.901	0.68 (0.37–1.25)	0.212
Parents married/living together								
No (reference)								
Yes	0.86 (0.55–1.35)	0.517	0.67 (0.29–1.55)	0.341	0.99 (0.39–2.5)	0.979	0.93 (0.36–2.37)	0.874
Previous Year Low Grades	1.11 (0.92–1.34)	0.268	1.29 (0.89–1.86)	0.180	**1.83 (1.20–2.79)**	**0.006**	**1.41 (1.02–1.95)**	**0.040**
Mother and/or father unemployed								
No (reference)								
Yes	0.86 (0.61–1.21)	0.369	0.6 (0.34–1.06)	0.080	0.5 (0.22–1.11)	0.088	**0.44 (0.21–0.91)**	**0.028**
Siblings								
No (reference)								
Yes	0.98 (0.62–1.57)	0.943	2.16 (0.87–5.4)	0.098	1.07 (0.46–2.52)	0.874	0.86 (0.42–1.78)	0.687
Father’s age	1.01 (0.96–1.06)	0.688	1.01 (0.95–1.08)	0.696	0.96 (0.87–1.05)	0.326	0.99 (0.93–1.06)	0.858
Mother’s age	1.02 (0.97–1.08)	0.353	**1.09 (1.01–1.17)**	**0.028**	1.01 (0.93–1.1)	0.827	1.01 (0.94–1.09)	0.708

+—Odds Ratio (95% Confidence Interval).

**Table 7:** Results from multiple logistic regression analysis with clinical internalizing problems as the dependent variable *for Romania.* Bold cells denote statistically significant associations.

	Internalizing Problems Normal/Borderline vs. Clinical	
	OR (95% CI) +	*p*
Gender		
Female (reference)		
Male	**3.20 (1.26–8.13)**	**0.015**
Age		
14–15.9 (reference)		
16–17.9	1.14 (0.48–2.73)	0.757
Parental educational level		
Low/Middle (reference)		
High	0.43 (0.16–1.19)	0.103
Parents married/living together		
No (reference)		
Yes	0.69 (0.15–3.15)	0.624
Previous Year Low Grades	1.20 (0.73–1.98)	0.474
Mother and/or father unemployed		
No (reference)		
Yes	1.22 (0.53–2.82)	0.637
Siblings		
No (reference)		
Yes	0.67 (0.24–1.83)	0.427
Father’s age	**1.06 (1.00–1.13)**	**0.041**
Mother’s age	0.97 (0.88–1.06)	0.498

+—Odds Ratio (95% Confidence Interval).

**Table 8:** Results from multiple logistic regression analysis with clinical internalizing problems (in all subscales and in total) as the dependent variable *for The Netherlands*. Bold cells denote statistically significant associations.

	Internalizing Problems Normal/Borderline vs. Clinical		Anxious/Depressed Normal/Borderline vs. Clinical		Withdrawn/Depressed Normal/Borderline vs. Clinical		Somatic Complaints Normal/Borderline vs. Clinical	
	OR (95% CI) +	*p*	OR (95% CI) +	*p*	OR (95% CI) +	*p*	OR (95% CI) +	*p*
Gender								
Female (reference)								
Male	0.92 (0.51–1.66)	0.773	2.31 (0.88–6.04)	0.088	0.53 (0.15–1.84)	0.315	1.47 (0.65–3.33)	0.349
Age								
14–15.9 (reference)								
16–17.9	**2.07 (1.12–3.85)**	**0.022**	1.42 (0.60–3.38)	0.420	2.09 (0.61–7.15)	0.235	**5.10 (1.65–15.71)**	**0.005**
Parental educational level								
Low/Middle (reference)								
High	0.81 (0.42–1.58)	0.536	0.74 (0.29–1.89)	0.517	0.50 (0.14–1.73)	0.265	4.09 (0.94–17.89)	0.061
Parents married/living together								
No (reference)								
Yes	0.79 (0.36–1.72)	0.539	1.58 (0.36–6.96)	0.539	0.57 (0.11–2.93)	0.498	0.42 (0.17–1.02)	0.056
Previous Year Low Grades	1.38 (0.91–2.09)	0.128	1.03 (0.52–2.02)	0.933	1.04 (0.34–3.20)	0.949	1.34 (0.66–2.73)	0.411
Mother and/or father unemployed								
No (reference)								
Yes	1.30 (0.67–2.52)	0.435	1.75 (0.54–5.69)	0.344	3.18 (0.72–14.16)	0.126	0.82 (0.3–2.23)	0.693
Siblings								
No (reference)								
Yes	2.03 (0.44–9.46)	0.362	1.84 (0.15–22.36)	0.627	0.45 (0.07–2.88)	0.393	2.21 (0.29–16.81)	0.437
Father’s age	1.00 (0.94–1.07)	0.952	1.06 (0.96–1.16)	0.223	1.01 (0.88–1.16)	0.868	0.95 (0.81–1.11)	0.503
Mother’s age	0.99 (0.9–1.09)	0.822	0.89 (0.77–1.02)	0.084	0.93 (0.81–1.07)	0.297	0.94 (0.81–1.1)	0.454

+—Odds Ratio (95% Confidence Interval).

## Data Availability

Data available upon request.
